# Candida albicans enriched in orthodontic derived white spot lesions and shaped focal supragingival bacteriome

**DOI:** 10.3389/fmicb.2023.1084850

**Published:** 2023-01-24

**Authors:** Hao Yang, Yansong Ma, Xianju Xie, Hongmei Wang, Xiaowei Li, Dongyu Fang, Yuxing Bai

**Affiliations:** Department of Orthodontics, School of Stomatology, Capital Medical University, Beijing, China

**Keywords:** microbial community, white spot lesion, fixed orthodontic treatment, *Candida albicans*, 16S rRNA gene sequencing

## Abstract

White spot lesions (WSLs) are common enamel infectious diseases in fixed orthodontic treatment, which might attribute to the dysbiosis of oral microbiome. However, the correlation of *Candida albicans* with oral bacteriome in WSLs still remains unrevealed. This study investigated the carriage of *C. albicans* and how it shaped the bacterial community in disease or healthy supragingival plaque, to explore the potential role of interkingdom interaction in orthodontic WSLs. In this study, 31 patients with WSLs (WSLs) and 23 healthy patients (Health) undergoing fixed orthodontic treatment were enrolled. The supragingival microbiota in both groups were determined using 16S rRNA gene sequencing. Colonization and abundance of *C. albicans* in the plaque were determined *via* culture-dependent and -independent methods. Among WSLs patients, the correlation of *C. albicans* and bacteriome was analyzed under QIIME2-based bioinformatics and Spearman’s correlation coefficient. The raw reads were deposited into the NCBI Sequence Read Archive (SRA) database (Accession Number: SRP404186). Significant differences in microbial diversity as well as composition were observed between WSLs and Health groups. *Leptotrichia* remarkably enriched in the WSLs group, while *Neisseria* and *Cardiobacterium* significantly enriched in the Health group. In addition, 45% of WSLs patients were *C. albicans* carriers but none in patients without WSLs. Among all WSLs patients, beta diversity and microbial composition were distinguished between *C. albicans* carriers and non-carriers. In *C. albicans* carriers, *Corynebacterium matruchotii* and *Streptococcus mutans* significantly enriched whereas *Saccharibacteria_TM7_G-1* significantly depleted. The abundance of *C. albicans* was positively associated with bacteria such as *Streptococcus mutans*, while the negative correlation was detected between *C. albicans* and several bacteria such as *Cardiobacterium hominis* and *Streptococcus sanguinis.* Our study elucidated the distinguished supragingival plaque microbiome between orthodontic patients with and without WSLs. *C. albicans* frequently existed and enriched in orthodontic derived WSLs. The carriage of *C. albicans* shape plaque bacterial community in demineralized lesions and might play roles in WSLs pathogenesis.

## 1. Introduction

White spot lesions (WSLs) are milky white opaque areas around brackets on the labial surface of teeth in fixed orthodontics. These lesions are caused by subsurface porosity from enamel demineralization, which has a greater risk of progressing to severe cavities than sound enamel (Enaia et al., 2011). As a frequently diagnosed side-effect, WSLs have a considerably high incidence in orthodontic treatment, even reported to occur in up to 60.9% of patients (Zheng et al., 2016). It is generally believed that lesions will recover by natural remineralization after bracket removal. However, mineral re-uptake on the surface layer through saliva has little improvement on the aesthetic and structural properties of enamel. Therefore, WSLs seriously jeopardize the dental hard tissue and negatively affect patient satisfaction (Travess et al., 2004).

Multiple factors attribute to the development of WSLs, such as microorganisms, diet, and local micro-environment. Among them, microorganisms play the most important role (Tang et al., 2022). With the placement of orthodontic appliances, biofilms can easily accumulate around brackets, particularly in patients with poor oral hygiene (Badea et al., 2019). An imbalanced bacterial community can be derived from a high-carbohydrate diet, thus leading to the enrichment of acidogenic and aciduric bacteria (Valm, 2019; Da Costa Rosa et al., 2021). Cariogenic bacteria like *Streptococcus mutans* (*S. mutans*) and *Leptotrichia wadei* could be isolated and cultivated from the enamel lesions of WSLs patients in previous studies (Andrucioli et al., 2017; KarabekİRoĞLu et al., 2017; Lee et al., 2021).

In recent years, the profile of oral microbiome and its change in fixed orthodontics have attracted more attention due to the advancement of high-throughput techniques (Sadeq et al., 2015; Sun et al., 2018). Through microbiological analysis of saliva and supragingival plaque samples, previous studies proposed that the oral microbiota was changed during the process of orthodontic treatment, and the plaque accumulation was boosted by the orthodontic appliance (Guo et al., 2021). With the progress of treatment, some caries-related bacteria increased, but the genus generally associated with a healthy status, such as *Rothia*, was decreased (Koopman et al., 2015). However, most studies mainly observed the microbiome change from the start of orthodontic treatment but rarely mentioned how the microbiome changed from healthy to the occurrence of typical WSLs. The difference in microbiome characteristics in supragingival plaque from orthodontic patients with or without WSLs has not been well reported. Hence, exploring the supragingival microbiome between healthy and disease status may attribute to a better understanding of the pathogenesis of WSLs.

In addition to bacteria, fungi were much less abundant but a non-negligible kingdom in the oral microbiome, which have been widely reported in multiple oral diseases (Dağistan et al., 2009; Peleg et al., 2010; Xu et al., 2013). *Candida albicans* (*C. albicans*) could be isolated in dental caries diseases such as early childhood caries (ECC) and root caries (Valm, 2019; Chen Y. et al., 2021). In these caries lesions, *C. albicans* had active interkingdom interactions with bacteria like *S. mutans*, which synergistically contribute to the development of dental caries (Valm, 2019; Ev et al., 2020). It was reported that *C. albicans* had a positive correlation with *S. mutans* in ECC-related biofilm (Bachtiar and Bachtiar, 2018). *S. mutans*-secreted GTFB binds to the mannan layer of *C. albicans* to promote extracellular matrix formation and their co-existence within biofilms in children with ECC (Hwang et al., 2017). Previous studies have investigated the correlation between *C. albicans* and oral bacteriome in ECC, results also showed that *C. albicans* had the strongest positive correlation with *S. mutans* (Yang et al., 2022). Besides *S. mutans*, the presence of oral *C. albicans* was associated with a highly acidogenic and acid-tolerant bacterial community in ECC, with an increased abundance of the genera such as *Streptococcus*, *Lactobacillus*, and *Scardovia* (Xiao et al., 2018). In addition, *C. albicans* was positively correlated with several caries-associated species, such as *Actinomyces sp. ICM58*, *Actinomyces sp. oral taxon 172*, and *Scardovia wiggsiae* (Baraniya et al., 2020). However, to date there was no study reporting whether *C. albicans* would be enriched in orthodontic WSLs. How *C. albicans* shape bacterial communities and interact with bacteriome in the WSLs disease sites was also unrevealed.

Therefore, both *C. albicans* and bacterial communities were investigated in this study. Supragingival bacteriomes were distinguished in orthodontic on-going patients with or without WSLs. In addition, the prevalence of *C. albicans* and its association with oral bacteria was studied. Three null hypotheses were proposed: (1) there were no significant differences in supragingival plaque microbiome between orthodontic patients with and without WSLs; (2) *C. albicans* would not enrich in patients with WSLs; and (3) The existence of *C. albicans* could not shape the bacterial composition and correlation in local supragingival plaque community.

## 2. Materials and methods

### 2.1. Patient recruitment

This study was approved by the Ethical Committee of Beijing Stomatological Hospital (No. CMUSH-IRB-KJ-PJ-2022-11). The patients provided their written informed consent to participate in this study. A total of 54 patients (aged 14.25 ± 1.92 years) who underwent fixed orthodontic treatment within 6–12 months were recruited from the department of orthodontics, Beijing stomatological hospital. Patients who had WSLs in anterior teeth or premolars area evaluated by averaged enamel decalcification index (EDI) >0.12 (Robertson et al., 2011) were allocated to the “WSLs group” (*n* = 31). Otherwise, patients without detectable WSLs were assigned to the “Health group” (*n* = 23). All included subjects were periodontal healthy, with no attachment loss, probing depth (PD) ≤3 mm, and gingival index (GI) ≤1. Clinical parameters were recorded by the same well-trained dentist. Subjects were excluded from this study if they had untreated caries in the whole dentition, enamel hypoplasia, tetracycline-stained teeth, dental fluorosis, or other oral and systemic diseases. Subjects who used antibiotics or any other medications that might affect the oral microbiota in the past 3 months were also excluded.

### 2.2. Sample collection

Participants refrained from any type of oral hygiene for 12 h, as well as diet intake for at least 2 h before sample collection. The supragingival plaqueswere amplified from the extracted from the labial surfaces of all anterior teeth and premolars around brackets were collected by scraping them with a sterilized periodontal curette. Samples in the WSLs group were collected from the enamel surfaces with WSLs, while samples grouped as Health were collected from the sound enamel sites. Scraped plaque sample was resuspended into a 1.5 mL microcentrifuge tube containing 900 μL sterilized TE buffer (10 mM Tris–HCl, pH 8.0, 1 mM EDTA) and then immediately transferred to the lab on ice. Each sample was vortexed for 20 s and mixed thoroughly by pipetting up and down 10 times, then divided into 3 equal aliquots for 300 μL per tube. Among the 3 aliquots, one aliquot of the sample was centrifuged and resuspended in 1 mL sterilized PBS supplemented with 20% glycerol for further *Candida* culture and identification. The other two aliquots were used for further DNA extraction. All three tubes were stored at −80°C before use.

### 2.3. DNA purification, 16S rRNA sequencing, and processing

For each sample, one aliquot tube resuspended in TE buffer was processed for DNA extraction and bacterial 16S rRNA sequencing. After thawing at room temperature, samples were firstly mechanically disrupted by 0.1 mm glass beads for 2 cycles, 30 s/cycle (Fastprep-24 5G, MP Biomedical, CA, USA). Total genomic DNA was then extracted using a commercial bacterial genome DNA extraction kit following the manufacturer’s instruction (FastDNA^®^ Spin Kit for Soil, MP Biomedicals, CA, USA). The quality and quantity of eluted DNA were measured by a spectrophotometer (NanoDrop 2000, Thermo Fisher Scientific, MA, USA), then stored at –80°C for further use. Bacterial 16S rRNA gene fragments (V1-V3) were amplified from the extracted DNA using specific primers 27F (5′-AGAGTTTGATCCTGGCTCAG-3′) and 533R (5′-TTACCGCGGCTGCTGGCAC-3′). The sample DNA library was finally obtained and constructed by 2 × 300 bp paired-end (PE) sequencing on the Illumina MiSeq platform using PE300 chemical at Majorbio Bio-Pharm Technology Co. Ltd. (Majorbio, Shanghai, China). The raw reads were deposited into the NCBI Sequence Read Archive (SRA) database (accession number: SRP404186).

After demultiplexing the adapter and primer from the raw data, the sequences were merged with FLASH (v1.2.11) and quality filtered with fastp (0.19.6) (Magoc and Salzberg, 2011; Chen et al., 2018). To obtain high-quality data and improve the accuracy of subsequent bioinformation analysis, sequences were de-noised using DADA2 plugin in the QIIME2 pipeline with recommended parameters, which obtained single nucleotide resolution based on error profiles within samples (Callahan et al., 2016; Bolyen et al., 2019). High-resolution taxonomic assignment of ASVs yield by DADA2 was performed using the Naive bayes consensus taxonomy classifier implemented in QIIME2 and established by alignment to the HOMD database (v15.2) (Chen et al., 2010).

### 2.4. Detection of *C. albicans*

Colonization of *C. albicans* was checked from all samples in both WSLs and Health groups. Samples stored in PBS with 20% glycerol were plated on CHROMagar *Candida* selective agar plates (Becton Dickinson & Co., Franklin Lakes, NJ, USA), and incubated at 37°C for 72 h (Pusateri et al., 2009; Ali et al., 2017). Colonies cultured by CHROMagar plate were preliminarily identified according to the color of the colony (i.e., *C. albicans* showed green colonies). Then, colonies were collected with sterilized inoculation loops and transferred to new tubes for further DNA extraction and rDNA ITS sequence analyses (Thiyahuddin et al., 2019).

After mechanical disruption with 0.5 mm glass beads, an Epicenter MasterPure DNA purification kit (Lucigen Corporation, Middleton, WI, USA) was used to purify the total genomic DNA of each sample. Identification of yeast species was carried out by PCR amplification and rDNA ITS sequence analyses. The PCR reactions were performed using ABI GeneAmp PCR System 9700 (Eppendorf Mastercycler gradient, HH, DE). A total of 2 × Taq PCR Mastermix (KT201, Tiangen Biotechnologies, Beijing, China) was used in PCR amplification, and ITS4/ITS5 primers were used (Supplementary Table 1; White et al., 1990). Each 50 μL PCR reaction system contained 17 μL sterile water, 25 μL 2 × Taq PCR Mastermix, 4 μL template DNA, and 2 μL each of forward and reverse ITS primers. PCR was performed as the following program: 95°C for 5 min; followed by 35 cycles of the following steps: denaturation at 95°C for 30 s; annealing at 55°C for 30 s; extension at 72°C for 1 min; and final extension step at 72°C for 10 min. PCR products were sent to the Beijing genomics institution for the identification of yeast species. After the sequences obtained by sequencing were assembled and corrected, blast software was used to compare the sequences in the GenBank database, and ITS sequences of related strains with high similarity were selected. The existence of *C. albicans* in the sample was double confirmed by: (1) the presence of colonies on CHROMagar plate; and (2) confirmation by ITS sequencing.

### 2.5. Quantification of *C. albicans*

To quantify the abundance of *C. albicans* in the samples, a droplet digital polymerase chain reaction (ddPCR) was performed. The total genomic DNA of the other aliquot of the supragingival plaque sample was extracted and purified as mentioned in section “2.4 Detection of *C. albicans*.” The SAP gene (Supplementary Table 1) of *C. albicans* was amplified and the ddPCR reactions were performed by the QX200 Droplet Digital PCR system (BioRad, Hercules, CA, USA). Each 20 μL ddPCR reaction system contained 8.6 μL sterile water, 10 μL 2 × ddPCR Supermix for EvaGreen, 1 μL template DNA, and 0.2 μL each of forward and reverse SAP primers. The mixture and droplet generation oil was added into DG8 Cartridges, and droplets were generated through the QX200 Droplet Generator (BioRad, Hercules, CA, USA). Next, the droplets were transferred to a 96-well PCR plate and sealed with PCR Plate Sealer (PX1, BioRad, Hercules, CA, USA). ddPCR was performed as the following program: 95°C for 10 min, followed by 40 cycles of 95°C for 30 s and 59.5°C for 80 s, and 1 cycle of 98°C for 10 min. Then, the PCR plate was put into a Droplet Reader to detect fluorescence (QX200, BioRad, Hercules, CA, USA). Finally, Quantalife Software 1.7.4 were used to analyze the data.

### 2.6. Statistical analyses

For microbiome sequencing data, the non-parametric Wilcoxon rank-sum test was used to identify ASVs in QIIME2. Principal Coordinate Analysis (PCoA) was applied to compare beta diversity between groups and the statistical significance was evaluated by the ADONIS test. Chao1, ACE, Simpson and Shannon indices were used to compare alpha diversity. Differences in taxonomic composition, and community function were evaluated using the Wilcoxon rank-sum test. Metastasis analysis was employed to identify bacteria with significant differences between groups at different classification levels based on false discovery rate (FDR). Indicated taxa enriched in different groups were determined by linear discriminant analysis (LDA) effect size (LEfSe). The threshold for distinguished logarithmic LDA score was set to 3.5.

Pearson’s chi-squared test was used to compare the detection rate of *C. albicans* between WSLs and the Health group. The Kruskal-Wallis test was used to compare the absolute abundance of *C. albicans* in different groups. Spearman’s correlation coefficient was applied to examine the correlations of the abundance of *C. albicans*, bacterial taxa, and EDI. A value of two-tailed *P* < 0.05 was considered statistically significant.

## 3. Results

### 3.1. The supragingival microbiome between WSLs and health were significantly different

In this research, patients with typical WSLs or without demineralization were recruited to study their microbial differences under fixed orthodontic treatment (Table 1). We first compared the characteristics of the supragingival microbiome between both groups *via* amplicon sequencing. A total of 3,779,993 raw reads were obtained from the 54 samples. By quality filtration, 2,728,802 optimized sequences were got, and the mean sequence length was 483 bp. After de-noised using the DADA2 plugin in the QIIME2 pipeline with recommended parameters, finally, a total of 407,413 sequences were obtained. Taxonomically classified ASVs were associated with 11 phyla, 20 classes, 28 orders, 42 families, 81 genera, and 297 species from all samples. Among all annotated genera, 63 genera were uniformly shared by both groups. Twelve genera were uniquely detected in the Health group while 6 genera were in the WSLs group (Supplementary Figure 1A).

**TABLE 1:** Means (standard deviation) of demographic data in this study.

Groups	Age	Duration of treatment months	EDI	GI	PD
WSLs (*n* = 31)	14.25 (1.62)	9.04 (1.70)	0.42 (0.22)	0.31 (0.15)	1.71 (0.11)
Health (*n* = 23)	14.25 (2.26)	9.06 (1.71)	0.00 (0.00)	0.29 (0.14)	1.70 (0.10)

**FIGURE 1:**
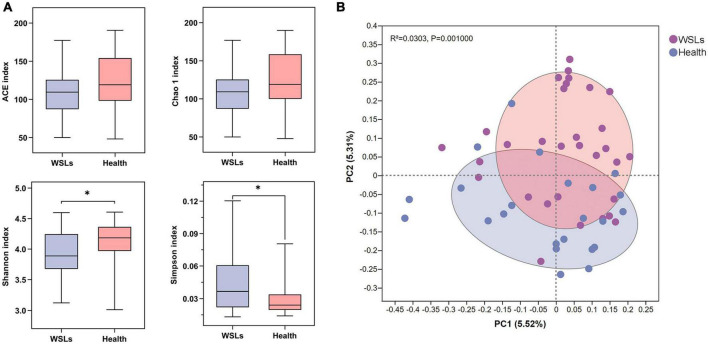
Comparisons of microbial diversities between WSLs and health group. **(A)** Alpha diversity was evaluated by indices of ACE, Chao1, Shannon, and Simpson. Simpson and Shannon indices showed significantly higher diversity in the microbial community of health group (*P < 0.05). **(B)** Beta diversity was calculated *via* the comparison of principal coordinates analysis (PCoA) of both groups. Each dot represents one sample. The communities in WSLs group tended to cluster apart from the communities in health group (*P* < 0.05).

To determine whether differences existed between patients with or without WSLs, alpha and beta diversity in both groups were analyzed (Figure 1). Simpson and Shannon indices showed that significantly higher community diversity in the Health group was found (*P* < 0.05). No significant difference was detected in richness by Chao 1 and ACE indices. In addition, we analyzed the overall bacterial community composition using PCoA plots. Results showed a significant difference in beta diversity (*P* < 0.05), revealing that most samples obtained from WSLs remarkably clustered away from those obtained from healthy teeth.

**FIGURE 2:**
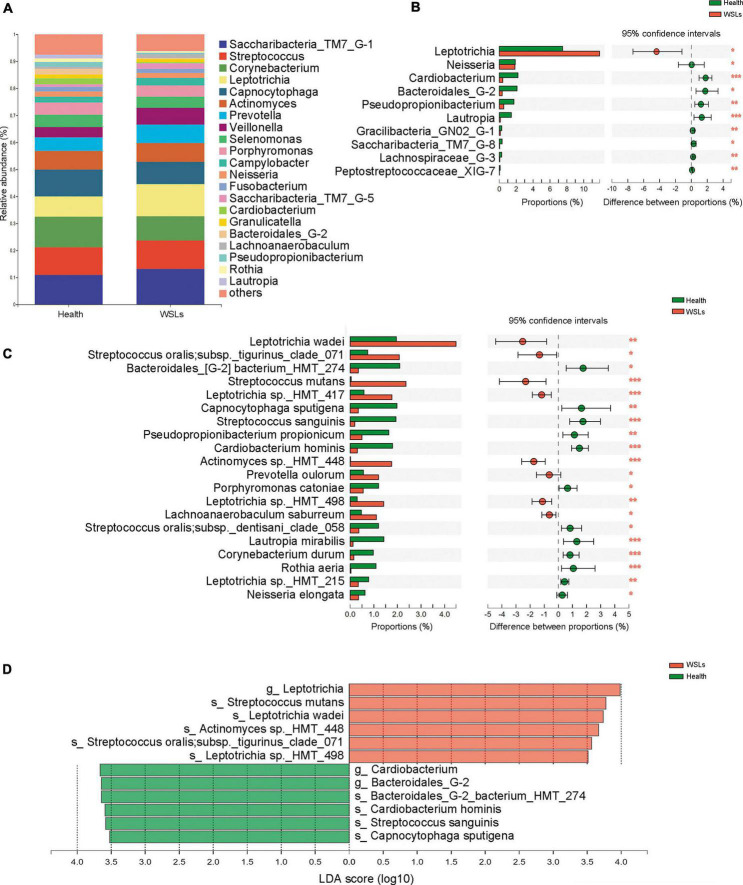
Comparison of microbial composition between WSLs and health group. **(A)** Relative abundances of the core genera (relative abundance >1.0%) in WSLs and health groups. “Others” represented all the genera less than 1%. **(B)** The most significantly different 10 genera in proportion between WSLs and health group were plotted (ranked by relative abundance). *Leptotrichia* was dominant in WSLs lesions while the others enriched in health group. **(C)** The top 20 species showed significant differences between WSLs and health group (ranked by relative abundance). **(D)** LEfSe analysis was performed between both groups at genus and species levels. Significantly differentiated distributed genera and species were displayed as the LDA score >3.5 (*P < 0.05, **P < 0.01, ***P < 0.001).

To further identify the distinct microbial composition between WSLs and healthy niches, relative abundance in phylum, genera, and species taxonomic levels was focused. In all phenotypes, the dominant phyla were *Firmicutes*, *Bacteroidetes*, *Actinobacteria*, *Saccharibacteria_TM7*, *Fusobacteria*, and *Proteobacteria* (Supplementary Figure 2A). The core genera (relative abundance >1.0%) in both groups were plotted in Figure 2A. Among them, the most dominant 10 genera were *Saccharibacteria_TM7_G-1*, *Streptococcus*, *Corynebacterium*, *Leptotrichia*, *Capnocytophaga*, *Actinomyces*, *Prevotella*, *Veillonella*, *Selenomonas*, and *Porphyromonas*. Compared with the Health group, *Leptotrichia* and *Prevotella* were relatively elevated in the WSLs group. *Leptotrichia* also showed significance in the WSLs group based on the analysis of the Wilcoxon rank-sum test and LDA score (*P* < 0.05). Besides, compared with the WSLs group, *Neisseria*, *Cardiobacterium, Bacteroidales G-2*, *Pseudopropionibacterium*, and *Lautropia* were significantly enriched in the Health group (*P* < 0.05) (Figure 2B).

At the species level, some *Streptococcus spp.* displayed prominently difference in abundance (Figures 2C, D), despite no statistical significance being examined in the genera *Streptococcus* between WSLs and Health. As the member of the mitis group (MGS), *Streptococcus sanguinis* was abundant in the Health group (*P* < 0.05). On the contrary, *S. mutans*, considered a contributor to the etiology of dental caries, was more abundant in the WSLs group (*P* < 0.05) (Simón-Soro et al., 2014). Moreover, *Leptotrichia wadei*, *Leptotrichia sp._HMT_417*, *Actinomyces sp._HMT_448*, *Prevotella oulorum*, and *Leptotrichia sp._HMT_498* were also enriched in the WSLs group (*P* < 0.05). In addition, *Bacteroidales_G-2 bacterium_HMT_274*, *Capnocytophaga sputigena*, *Pseudopropionibacterium propionicum*, *Cardiobacterium hominis*, *Porphyromonas catoniae*, *Lautropia mirabilis, Corynebacterium durum*, *Rothia aeria*, *Leptotrichia sp._HMT_215*, and *Neisseria elongata* had a richer abundance in Health group (*P* < 0.05).

### 3.2. *C. albicans* colonization and load was remarkably higher in WSLs group

**FIGURE 3:**
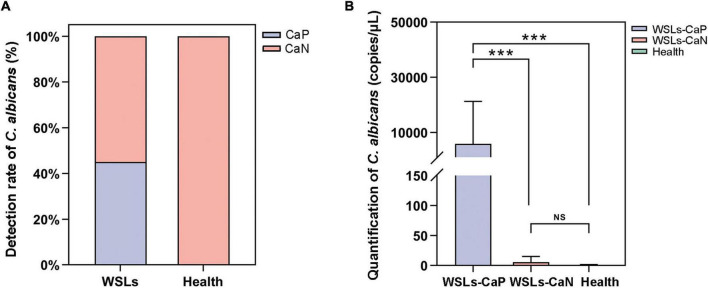
*Candida albicans* infection in the supragingival plaque. **(A)** The positive rate of *C. albicans* in WSLs and health groups. Positive result of *C. albicans* infection was determined by inoculation once the colonies emerged on CHROMAgar. Among all WSLs patients, 45% were *C. albicans* positive, but none in the health group. CaP: *C. albicans* positive; CaN: *C. albicans* negative. **(B)** Quantification of *C. albicans* in supragingival plaque among WSLs-CaP, WSLs-CaN, and health groups. *C. albicans* (copies/uL) in WSL-CaP group was remarkably higher than WSL-CaN and health groups (NS: no significance, ***P < 0.001).

The colonization of *C. albicans* in the plaque was first examined by sample inoculation in CHROMagar, and further molecular identification of colonies that emerged on CHROMagar was carried out by ITS sequencing. Interestingly, no colony emerged on CHROMagar in the Health group, while 14 samples showed green colonies among 31 WSLs samples (Supplementary Figure 3A). In further species identification by PCR and ITS sequencing, colonies from 14 samples were all *C. albicans*. Accordingly, as shown in Figure 3A, the detection rate of *C. albicans* in WSLs was significantly higher than that in the Health group (χ2 = 14.586, *P* < 0.01). This result indicated that the colonization of *C. albicans* was more likely to occur in demineralization lesions rather than in healthy niches.

Next, samples from the WSLs group were further divided into two subgroups, namely the *C. albicans* cultivation positive subgroup (WSLs-CaP) and the *C. albicans* cultivation negative subgroup (WSLs-CaN). The absolute abundance of *C. albicans* was determined by ddPCR analysis. All three groups were performed with ddPCR although most samples didn’t have any colony come up. As shown in Figure 3B, the abundance of *C. albicans* in the WSLs-CaP group was significantly higher than in WSLs-CaN as well as Health group (*P* < 0.01), which verified those samples could be inoculated with *C. albicans* colonies indeed had much higher load than colony-free samples. In addition, no significant difference was found between WSLs-CaN as well as Health groups (*P* > 0.05).

### 3.3. Enrichment of *C. albicans* affects the composition of supragingival microbiome in WSLs

**FIGURE 4:**
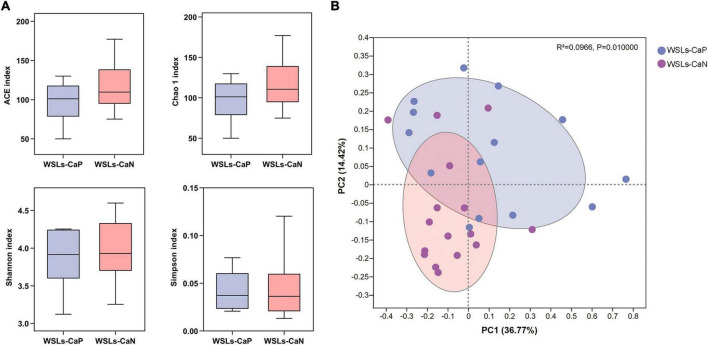
Comparisons of microbial diversities between WSLs-CaP and WSLs-CaN groups. **(A)** Alpha diversity was evaluated by indices of ACE, Chao1, Shannon, and Simpson. No significance was detected in all indices. **(B)** Beta diversity was calculated *via* the comparison of principal coordinates analysis (PCoA). Each dot represents one sample. The communities in WSLs-CaP group tended to cluster apart from the communities in WSLs-CaN group (*P* < 0.05).

We next proposed whether the enrichment of *C. albicans* could affect the bacterial composition in the plaque. Following the results above, analysis was performed between WSLs-CaP and WSLs-CaN subgroups in this section. Among all annotated genera, 57 genera were uniformly shared by both groups. One genus was uniquely detected in the WSLs-CaP group while 11 genera were in the WSLs-CaN group (Supplementary Figure 1B). As shown in Figure 4, there was no significant difference in alpha diversity. For beta diversity, the PCoA of the two principal components accounted for 36.77 and 14.42% of the total variation, respectively, suggesting the separation between the two subgroups was significant (*R*^2^ = 0.0966, *P* < 0.05).

**FIGURE 5:**
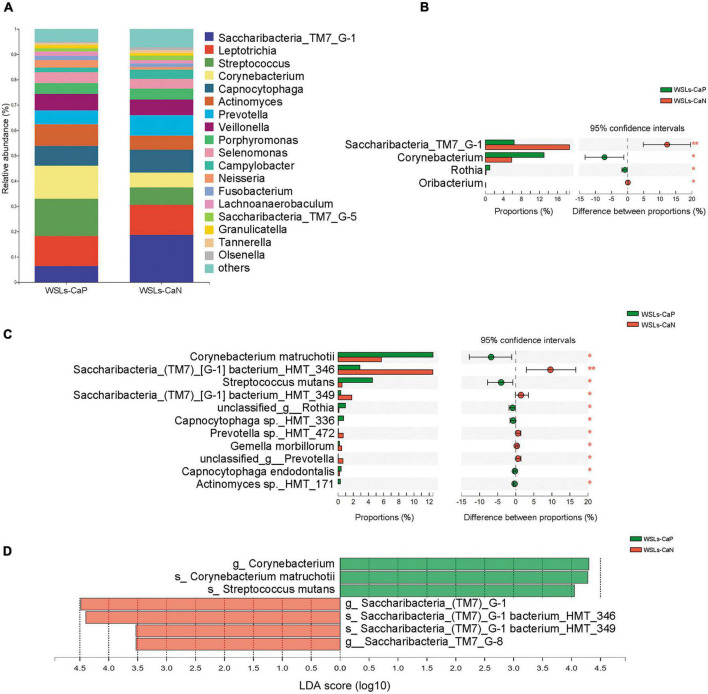
Comparison of microbial composition between *Candida albicans* carriers and non-carriers in WSLs patients. **(A)** Relative abundances of the core genera (relative abundance >1.0%) in WSLs-CaP and WSLs-CaN groups. “Others” represented all the genera less than 1%. **(B)** Significantly differentiated genera between WSLs-CaP and WSLs-CaN groups. *Saccharibacteria_TM7_G-1* was much enriched in plaque without *C. albicans* infection, while *Corynebacterium*, *Rothia* and *Oribacterium* were enriched in *C. albicans* positive group. **(C)** Significantly different species examined between WSLs-CaP and WSLs-CaN group were displayed. In *C. albicans* carriers, *Corynebacterium matruchotii* and *Streptococcus mutans* significantly enriched, whereas several species such as *Saccharibacteria_TM7_G-1 bacterium_HMT_346* and *Saccharibacteria_TM7_G-1 bacterium_HMT_349* significantly enriched in *C. albicans* non-carriers. **(D)** LEfSe analysis was performed between WSLs-CaP and WSLs-CaN groups at the genus and species levels. Significantly differentiated distributed genera and species were displayed as the LDA score >3.5 (*P < 0.05, **P < 0.01).

In all phenotypes, the dominant phyla were also *Firmicutes*, *Bacteroidetes*, *Actinobacteria*, *Saccharibacteria_TM7*, *Fusobacteria*, and *Proteobacteria* (Supplementary Figure 2B). The relative abundance of core genera (relative abundance >1.0%) were compared to illustrate whether *C. albicans* affect the microbial composition (Figure 5A). Compared to the WSLs-CaN subgroup, *Corynebacterium* and *Rothia* were the predominant genera in the WSLs-CaP subgroup (*P* < 0.05). *Corynebacterium matruchotii* and *Streptococcus mutans* had much higher abundance in the WSLs-CaP subgroup (*P* < 0.05). On the contrary, *Saccharibacteria_TM7_G-1* significantly enriched in the WSLs-CaN group (*P* < 0.05) with its two species *Saccharibacteria_TM7_G-1 bacterium_HMT_346* and *Saccharibacteria_TM7_G-1 bacterium_HMT_349* showing the same trend (*P* < 0.05) (Figures 5B–D). This interesting results suggested the existence of *C. albicans* might shape the bacterial community in WSLs disease condition.

### 3.4. Correlation between *C. albicans*, bacterial and EDI

**FIGURE 6:**
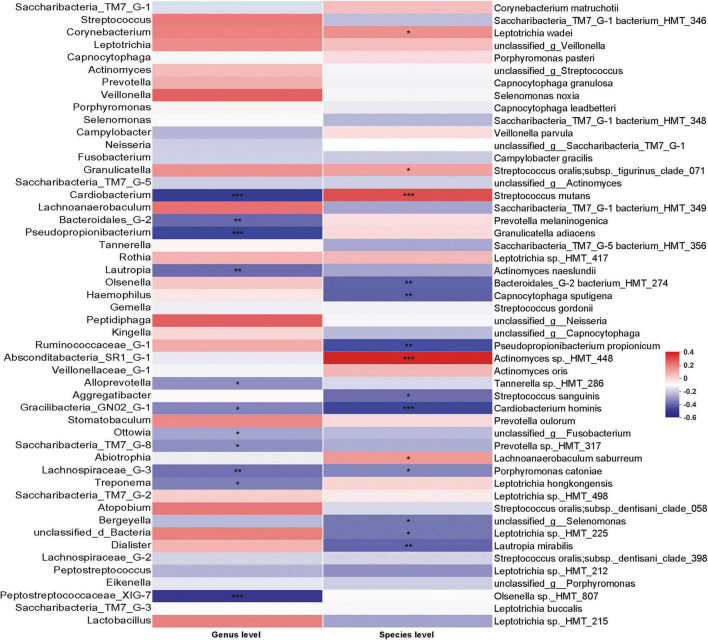
Correlation analysis between *Candida albicans* and bacteria based on Spearman correlation coefficient. The correlation between the quantification of *C. albicans* and the top 50 abundant bacteria genera and species in the rank of relative abundance was evaluated. At genus level, bacteria such as *Saccharibacteria_TM7_G-8* negatively associated with *C. albicans*. At species level, *Streptococcus mutans* had positive association with the load of *C. albicans*. Several species such as *Streptococcus sanguinis* and *Cardiobacterium hominis* had depleting trend in *C. albicans* enriched samples. Blocks in red showed positive correlation, while blocks in blue showed negative correlation. Color depth implicated the strength of correlation (*P < 0.05, **P < 0.01, ***P < 0.001).

Although the existence of *C. albicans* may change the bacterial community in dental plaque, the correlation between the abundance of *C. albicans* and bacterial changes was not clear. We thus performed Spearman’s rank correlation analysis to specifically examine the correlation between the abundance of *C. albicans* and bacterial taxa (Figure 6). *S. mutans*, which is the main contributor to dental caries, was positively correlated with *C. albicans*. In addition, bacteria showed significantly higher abundance in patients with WSLs, such as *Leptotrichia wadei*, *Actinomyces sp._HMT_448*, and *Lachnoanaerobaculum saburreum*, were also exhibited a positive correlation with *C. albicans*.

Interestingly, on the contrary, *S. sanguinis*, which can release H_2_O_2_ thereby inhibiting the growth of *S. mutans* and being prone to be detected on the healthy teeth surface, were negatively correlated with *C. albicans* (Kreth et al., 2009). In addition, some other bacteria showed significantly higher abundance in the Health group, such as *Cardiobacterium*, *Bacteroidales_G-2*, *Pseudopropionibacterium*, *Lautropia*, *Gracilibacteria_GN02_G-1*, *Saccharibacteria_TM7_G-8*, *Lachnospiraceae_G-3*, and *Peptostreptococcaceae_XIG-7* exhibited a negative correlation with *C. albicans*. At the species level, *Bacteroidales_G-2 bacterium_HMT_274*, *Capnocytophaga sputigena*, *Pseudopropionibacterium propionicum*, *Cardiobacterium hominis*, *Porphyromonas catoniae*, *Leptotrichia sp._HMT_225*, and *Lautropia mirabilis* exhibited a negative correlation with *C. albicans* as well.

To address whether the *C. albicans* abundance was correlated with the severity of EDI, we investigated a Spearman’s rank correlation between EDI and *C. albicans* abundance (Supplementary Figure 3B). The result showed that no significant correlation between EDI and *C. albicans* abundance in the WSLs group (*P* = 0.09). In other words, although *C. albicans* was significantly enriched in the WSLs group, it did not show a significant correlation with the severity of WSLs.

## 4. Discussion

White spot lesions in fixed orthodontics are demineralization opacities that occur on the enamel surface and induce irreversible effects on dental hard tissue. The process of demineralization is closely related to plaque accumulation from which the microbial structure and metabolism might change accordingly. To deepen the understanding of microbiological pathogenesis in the development of WSLs, this study firstly investigated the differences of the supragingival microbiome in orthodontic patients with or without visible WSLs. Our results demonstrated that great changes could be observed between healthy and disease status, and all the three null-hypotheses were rejected. Compared to patients with healthy enamel, the WSLs group had significantly lower alpha diversity and differentiated clustering features in the community, which was consistent with previous studies (Hurley et al., 2019; Schoilew et al., 2019). This might be due to the cariogenic microenvironment that acidogenic bacteria produced acidic metabolites and then decreased the local pH (Corralo et al., 2021). Inhibition of acid-non-resistant bacteria and the overgrowth of aciduric bacteria led to lower diversity in the plaque community.

In terms of the difference in microbial composition, our results undoubtedly showed that the abundance of *S. mutans* had a significant difference between both groups. This demonstrated the cohorts we selected were comparative and that the samples we collected were typical (Thompson and Pikis, 2012; Qudeimat et al., 2021). *Leptotrichia* was one of the predominant genera in the plaque from the WSLs niche, which was consistent with the previous report (Yun et al., 2019). *Leptotrichia* could actively metabolize carbohydrates with higher membrane transport capacity (Eribe and Olsen, 2017; Chen Y. et al., 2021). This plaque-enriched genus was also reported to be able to produce extracellular polysaccharides (EPS) through carbohydrate anabolism, as well as multiple cariogenic organic acids as its end product through catabolism. With such a higher abundance in supragingival plaque, *Leptotrichia* was very likely associated with the development of enamel demineralization (Eribe and Olsen, 2017). Moreover, two of its main species *Leptotrichia wadei* and *Leptotrichia sp._HMT_498* were also reported with increasing abundance in adolescents with active dental caries (Eriksson et al., 2017). *Leptotrichia wadei*, with significantly higher abundance in our patients with demineralization, was also regarded as one of the predictors for dental caries (Hurley et al., 2019; Chen J. et al., 2021). This implies that its richness in orthodontic patients might be a warning indicator of WSLs. Besides, we found *Actinomyces sp._HMT_448* enriched in the WSLs group, despite the abundance of *Actinomyces* spp. having almost no difference between both groups. *Actinomyces sp._HMT_448* was reported with vigorous metabolism of carbohydrates, and producing lactic acid at lower pH level (Eriksson et al., 2017; Corralo et al., 2021). Therefore, the accumulation of this species also needs to be paid attention to in orthodontic WSLs.

Some genera such as *Neisseria*, *Cardiobacterium*, *Bacteroidales* G-2, *Pseudopropionibacterium*, and *Lautropia* were found enriched on healthy sites but depleted in WSLs. These results were consistent with the previous studies (Glogauer et al., 2015; Chen Y. et al., 2021), which indicated these bacteria might be associated with the stability of a healthy microbiome. Some of these healthy associated members are enriched may be due to their intrinsic metabolic characteristics. *Lautropia* had a higher level of signal transduction, xenobiotic biodegradation, and metabolism, which was conducive to dental health (Al-Kamel et al., 2019; Chen Y. et al., 2021). *Neisseria* could degrade lactate to acetate and exhibit higher arginine metabolism, thus neutralizing the acidification of the dental plaque (Rosier et al., 2020). Patients without dental caries also reportedly had a higher abundance of *Lautropia* and *Neisseria* in their dental plaques, their predominant abundance could be regarded as beneficial to oral health (Rosier et al., 2020). Besides, *Pseudopropionibacterium propionicum* could actively metabolize amino acid, which might be conducive to the neutralization of acid in dental plaque (Chen Y. et al., 2021). In addition, although result showed no significant differences in the abundance of *Corynebacterium* in two groups, it was relatively elevated in the Health group (Figure 2A). Previous study proposed that *Corynebacterium* could raise the pH of dental plaque by utilizing organic acids produced by other microbes (Qudeimat et al., 2021). Also, it can shape symbiotic supragingival biofilm communities by having anfractuous interactions with health-associated bacteria (Treerat et al., 2020). Moreover, we observed completely opposite trends in the abundance distribution of *S. mutans* and *S. sanguinis* in both groups. As a member of the Mitis Group *Streptococcus*, *S. sanguinis*, enriched in the Health group, can release H_2_O_2_ thereby inhibiting the growth of *S. mutans* (Kreth et al., 2009).

*Candida* carriage in patients undergoing orthodontic treatment has raised the attention of orthodontists (Zheng et al., 2016; Alhamadi et al., 2017). However, to date, there was no study yet reporting the association between *C. albicans* and orthodontic-derived early caries. In addition to oral bacteria, we studied whether *C. albicans* were dissimilarly allocated in both groups. Interestingly, all colonies emerged on CHROMagar only from the WSLs group, suggesting patients with WSLs preferably had *C. albicans* infection. We further confirmed a much higher load of *C. albicans* carriage in WSLs sites by ddPCR quantification. As a technique with high sensitivity and accuracy, ddPCR can realize the absolute quantification of nucleic acids (Hindson et al., 2011). ddPCR divides reagents into tens of thousands of nanoliter or picoliter partitions by a microfluidic chip, and each droplet contains 0 or 1 DNA template. After polymerase chain reaction amplification and the detection of fluorescence, the target nucleic acids were calculated according to the number of positive droplets (Pinheiro et al., 2012). Furthermore, each droplet is an independent closed reaction environment, which can reduce the possibility of contamination between droplets (Wang et al., 2018). On the other hand, one of the disadvantages is that the operation of ddPCR is relatively complex. The ddPCR results also proved that *C. albicans* would be more possible to occur and accumulate in orthodontic patients who had enamel demineralization. Furthermore, we found that the enrichment of *C. albicans* could affect the bacterial composition in the supragingival dental plaque. Analysis between *Candida* carriers and non-carriers among WSLs patients showed the difference in beta-diversity was significant (*P* < 0.05), revealing that the microbial community obtained from *Candida* carriers remarkably clustered away from non-carriers.

The existence of *Candida* could affect the bacteriome composition, which has been reported in different habitats (Du et al., 2021). Intriguingly, in our study *S. mutans* was more predominant in *C. albicans* carriers rather than non-carriers among all WSLs patients. This result supported these two interkingdom species having close interactions and positive correlations. Their co-existence has been widely reported in caries disease models like ECC and root caries (Xiao et al., 2016). Moreover, their cariogenic co-pathogenesis has also been extensively investigated. Coaggregation of *C. albicans* and *S. mutans* was very important for their co-localization and biofilm formation on tooth surfaces (Metwalli et al., 2013; Xu et al., 2014). *C. albicans* could grow better in *C. albicans*-*S. mutans* dual-species biofilm than its mono-species biofilm under the stimulation of *S. mutans* (Arzmi et al., 2016). When pioneer colonizers (e.g., *S. mutans*) initially adhered to the teeth, the activation of glucosyltransferases (GTFs) increased, especially the *gtfB* gene. Among GTFs, GTF-I was encoded by the *gtfB* gene and used sucrose as a substrate to synthesize water-insoluble glucan, which could participate in sucrose-dependent adhesion between bacteria and enamel surface (Bowen and Koo, 2011; Gregoire et al., 2011). These further became the binding sites for subsequent colonizers like *C. albicans* (Gregoire et al., 2011). Conversely, the *Candida*-derived mannan, and β-glucan also provided binding sites for GTFB, and the structure of EPS could be affected by β-1,3-glucans as well (Falsetta et al., 2014; Xu et al., 2014). Our results showed this interkingdom interaction might also exist in orthodontic derived initial enamel caries, that is particularly noteworthy in fixed orthodontic treatment. However, their potential role in the development of orthodontic WSLs still required long-term observation of microbial change to confirm.

In this study, other bacteria such as *C. hominis*, *S. sanguinis*, and *L. mirabilis* showed negative correlation with *C. albicans*. Among them, *C. albicans* showed lower virulence in interaction with *S. sanguinis* (Do Rosário Palma et al., 2019). The adhesion ability and Young’s modulus of *C. albicans* also decreased after being antagonized by *S. sanguinis* bacteriocin (Ma et al., 2017). The intracellular protein of *S. sanguinis* has a significant inhibitory effect on *C. albicans* and its biofilm. In addition, the growth curve and morphology of *C. albicans* changed as well, leading to discoid depressions on the surface of fungal spores and mycelia (Ma et al., 2014). Besides *S. sanguinis*, however, other bacteria which were negatively related to *C. albicans* in this study were rarely reported in previous research, so further studies are needed.

Besides, the result showed that there was no significant correlation between the abundance of *C. albicans* and the severity of EDI in the WSLs group. It was known that obvious WSLs can occur within 6 months of orthodontic treatment (Lucchese and Gherlone, 2012). Patients undergoing fixed orthodontic treatment within 6 to 12 months were recruited in this study (Tufekci et al., 2011). However, with the prolonged treatment time, such as at the 24 months of treatment, the occurrence of WSLs would further increase (Khalaf, 2014). Previous studies had shown that compared with the healthy teeth in caries-free children, the dental plaque on the healthy teeth surface of caries-active children was more similar to that with enamel caries, and had a higher risk of dental caries (Richards et al., 2017). Therefore, it was speculated that the change of microorganisms was earlier than the occurrence and aggravation of WSLs. In addition, the sample size will also affect the correlation results. One of the limitations of this study was that the sample size was relatively small, it is necessary to further increase the sample size in future research.

## 5. Conclusion

In conclusion, the present study revealed significant differences in the supragingival plaque microbiome between orthodontic patients with and without WSLs. *C. albicans* was more frequently detected and enriched in the plaque with WSLs rather than in the healthy site. The existence of *C. albicans* could shape the bacterial composition in the supragingival plaque community. *C. albicans* have a certain interkingdom association with bacteriomes. This study further enhanced the understanding of WSLs from the perspective of the oral microbiome, which is conducive to the exploration of potential diagnostic approaches and interventions in orthodontic-induced WSLs.

## Data availability statement

The datasets presented in this study can be found in online repositories. The names of the repository/repositories and accession number(s) can be found below: https://www.ncbi.nlm.nih.gov/, SRP404186.

## Ethics statement

This study was approved by the Ethical Committee of Beijing Stomatological Hospital (No. CMUSH-IRB-KJ-PJ-2022-11). Written informed consent to participate in this study was provided by the participants’ legal guardian/next of kin.

## Author contributions

HY and YM performed the experiments, data analyses, and manuscript writing. XX contributed to the data analysis. HW analyzed the data and revised the manuscript. XL and DF helped complete the experiments. YM designed the study, proposed ideas, provided discussions, and advised on manuscript revise. YB conceived the experiments, supervised the experiment progress, and revised the manuscript. All authors contributed to the article and approved the submitted version.

## References

[B1] Alhamadi W. Al-Saigh R. J. Al-Dabagh N. N. Al-Humadi H. W. (2017). Oral *Candida* in patients with fixed orthodontic appliance: *In vitro* combination therapy. *Biomed. Res. Int.* 2017:1802875. 10.1155/2017/1802875 28685145 PMC5480024

[B2] Ali R. H. Luma T. A. Ali S. M. (2017). Molecular and genotypes identification of *C. albicans* isolated from children with diarrea in diyala province Iraq. *Int. J. Biotechnol. Res.* 7 1–10.

[B3] Al-Kamel A. Baraniya D. Al-Hajj W. A. Halboub E. Abdulrab S. Chen T. (2019). Subgingival microbiome of experimental gingivitis: Shifts associated with the use of chlorhexidine and N-acetyl cysteine mouthwashes. *J. Oral. Microbiol.* 11:1608141. 10.1080/20002297.2019.1608141 31275528 PMC6598494

[B4] Andrucioli M. C. D. Faria G. Nelson-Filho P. Romano F. L. Matsumoto M. A. N. (2017). Influence of resin-modified glass ionomer and topical fluoride on levels of *Streptococcus mutans* in saliva and biofilm adjacent to metallic brackets. *J. Appl. Oral. Sci.* 25 196–202. 10.1590/1678-77572016-0231 28403360 PMC5393540

[B5] Arzmi M. H. Alnuaimi A. D. Dashper S. Cirillo N. Reynolds E. C. McCullough M. (2016). Polymicrobial biofilm formation by *Candida albicans*, *Actinomyces naeslundii*, and *Streptococcus mutans* is *Candida albicans* strain and medium dependent. *Med. Mycol.* 54 856–864. 10.1093/mmy/myw042 27354487

[B6] Bachtiar E. W. Bachtiar B. M. (2018). Relationship between *Candida albicans* and *Streptococcus mutans* in early childhood caries, evaluated by quantitative PCR. *F1000Res* 7 1645–1660. 10.12688/f1000research.16275.2 30450201 PMC6221075

[B7] Badea M. E. Mesaroş A. Şuhani R. D. Cosma L. L. (2019). Current treatment modalities of orthodontically induced white spot lesions and their outcome-a literature review. *Med. Pharm. Rep.* 92 25–30. 10.15386/cjmed-1090 30957083 PMC6448498

[B8] Baraniya D. Chen T. Nahar A. Alakwaa F. Hill J. Tellez M. (2020). Supragingival mycobiome and inter-kingdom interactions in dental caries. *J. Oral. Microbiol.* 12:1729305. 10.1080/20002297.2020.1729305 32158514 PMC7048226

[B9] Bolyen E. Rideout J. R. Dillon M. R. Bokulich N. A. Abnet C. C. Al-Ghalith G. A. (2019). Reproducible, interactive, scalable and extensible microbiome data science using QIIME 2. *Nat. Biotechnol.* 37 852–857. 10.1038/s41587-019-0209-9 31341288 PMC7015180

[B10] Bowen W. H. Koo H. (2011). Biology of *Streptococcus mutans*-derived glucosyltransferases: Role in extracellular matrix formation of cariogenic biofilms. *Caries Res.* 45 69–86. 10.1159/000324598 21346355 PMC3068567

[B11] Callahan B. J. McMurdie P. J. Rosen M. J. Han A. W. Johnson A. J. A. Holmes S. P. (2016). DADA2: High-resolution sample inference from Illumina amplicon data. *Nat. Methods* 13 581–583. 10.1038/nmeth.3869 27214047 PMC4927377

[B12] Chen J. Kong L. Peng X. Chen Y. Ren B. Li M. (2021). Core microbiota promotes the development of dental caries. *Appl. Sci.* 11:3638. 10.3390/app11083638

[B13] Chen S. Zhou Y. Chen Y. Gu J. (2018). fastp: An ultra-fast all-in-one FASTQ preprocessor. *Bioinformatics* 34 i884–i890. 10.1093/bioinformatics/bty560 30423086 PMC6129281

[B14] Chen T. Yu W. H. Izard J. Baranova O. V. Lakshmanan A. Dewhirst F. E. (2010). The human oral microbiome database: A web accessible resource for investigating oral microbe taxonomic and genomic information. *Database* 2010:baq013. 10.1093/database/baq013 20624719 PMC2911848

[B15] Chen Y. Dou G. Wang D. Yang J. Zhang Y. Garnett J. A. (2021). Comparative microbial profiles of caries and black extrinsic tooth stain in primary dentition. *Caries Res.* 55 310–321. 10.1159/000517006 34247164

[B16] Corralo D. J. Ev L. D. Damé-Teixeira N. Maltz M. Arthur R. A. Do T. (2021). Functionally active microbiome in supragingival biofilms in health and caries. *Caries Res.* 55 603–616. 10.1159/000518963 34380135

[B17] Da Costa Rosa T. De Almeida Neves A. Azcarate-Peril M. A. Divaris K. Wu D. Cho H. (2021). The bacterial microbiome and metabolome in caries progression and arrest. *J. Oral. Microbiol.* 13:1886748. 10.1080/20002297.2021.1886748 34188775 PMC8211139

[B18] Dağistan S. Aktas A. E. Caglayan F. Ayyildiz A. Bilge M. (2009). Differential diagnosis of denture-induced stomatitis, *Candida*, and their variations in patients using complete denture: A clinical and mycological study. *Mycoses* 52 266–271. 10.1111/j.1439-0507.2008.01592.x 18643887

[B19] Do Rosário Palma A. L. Domingues N. De Barros P. P. Brito G. N. B. Jorge A. O. C. (2019). Influence of *Streptococcus mitis* and *Streptococcus sanguinis* on virulence of *Candida albicans*: *In vitro* and *in vivo* studies. *Folia. Microbiol*. 64 215–222. 10.1007/s12223-018-0645-9 30232727

[B20] Du Q. Ren B. He J. Peng X. Guo Q. Zheng L. (2021). *Candida albicans* promotes tooth decay by inducing oral microbial. *ISME. J.* 15 894–908. 10.1038/s41396-020-00823-8 33149208 PMC8026629

[B21] Enaia M. Bock N. Ruf S. (2011). White-spot lesions during multibracket appliance treatment: A challenge for clinical excellence. *Am. J. Orthod. Dentofacial. Orthop.* 140 e17–e24. 10.1016/j.ajodo.2010.12.016 21724067

[B22] Eribe E. R. K. Olsen I. (2017). Leptotrichia species in human infections II. *J. Oral. Microbiol.* 9:1368848. 10.1080/20002297.2017.1368848 29081911 PMC5646626

[B23] Eriksson L. Lif Holgerson P. Johansson I. (2017). Saliva and tooth biofilm bacterial microbiota in adolescents in a low caries community. *Sci. Rep.* 7:5861. 10.1038/s41598-017-06221-z 28724921 PMC5517611

[B24] Ev L. D. DamÉ-Teixeira N. Do T. Maltz M. Parolo C. C. F. (2020). The role of *Candida albicans* in root caries biofilms: An RNA-seq analysis. *J. Appl. Oral. Sci.* 28:e20190578. 10.1590/1678-7757-2019-0578 32348446 PMC7185980

[B25] Falsetta M. L. Klein M. I. Colonne P. M. Scott-Anne K. Gregoire S. Pai C.-H. (2014). Symbiotic relationship between *Streptococcus mutans* and *Candida albicans* synergizes virulence of plaque biofilms *in vivo*. *Infect. Immun.* 82 1968–1981. 10.1128/iai.00087-14 24566629 PMC3993459

[B26] Glogauer M. Chen L. Qin B. Du M. Zhong H. Xu Q. (2015). Extensive description and comparison of human supragingival microbiome in root caries and health. *PLoS One* 10:e0117064. 10.1371/journal.pone.0117064 25658087 PMC4319720

[B27] Gregoire S. Xiao J. Silva B. B. Gonzalez I. Agidi P. S. Klein M. I. (2011). Role of glucosyltransferase B in interactions of *Candida albicans* with *Streptococcus mutans* and with an experimental pellicle on hydroxyapatite surfaces. *Appl. Environ. Microbiol.* 77 6357–6367. 10.1128/aem.05203-11 21803906 PMC3187131

[B28] Guo R. Zheng Y. Zhang L. Shi J. Li W. (2021). Salivary microbiome and periodontal status of patients with periodontitis during the initial stage of orthodontic treatment. *Am. J. Orthod. Dentofacial. Orthop.* 159 644–652. 33608141 10.1016/j.ajodo.2019.11.026

[B29] Hindson B. J. Ness K. D. Masquelier D. A. Belgrader P. Heredia N. J. Makarewicz A. J. (2011). High-throughput droplet digital PCR system for absolute quantitation of DNA copy number. *Anal. Chem.* 83 8604–8610. 10.1021/ac202028g 22035192 PMC3216358

[B30] Hurley E. Barrett M. P. J. Kinirons M. Whelton H. Ryan C. A. Stanton C. (2019). Comparison of the salivary and dentinal microbiome of children with severe-early childhood caries to the salivary microbiome of caries-free children. *BMC Oral Health* 19:13. 10.1186/s12903-018-0693-1 30642327 PMC6332856

[B31] Hwang G. Liu Y. Kim D. Li Y. Krysan D. J. Koo H. (2017). *Candida albicans* mannans mediate *Streptococcus mutans* exoenzyme GtfB binding to modulate cross-kingdom biofilm development *in vivo*. *PLoS. Pathog.* 13:e1006407. 10.1371/journal.ppat.1006407 28617874 PMC5472321

[B32] KarabekİRoĞLu S. ÜNlÜ N. KÜÇÜKyilmaz E. ŞEner S. Botsali M. S. MalkoÇ S. (2017). Treatment of post-orthodontic white spot lesions with CPP-ACP paste: A three year follow up study. *Dent. Mater. J.* 36 791–797. 10.4012/dmj.2016-228 28835597

[B33] Khalaf K. (2014). Factors affecting the formation, severity and location of white spot lesions during orthodontic treatment with fixed appliances. *J. Oral. Maxillofac. Res.* 5:e4. 10.5037/jomr.2014.5104 24800054 PMC4007370

[B34] Koopman J. E. Van der Kaaij N. C. Buijs M. J. Elyassi Y. Van der Veen M. H. Crielaard W. (2015). The effect of fixed orthodontic appliances and fluoride mouthwash on the oral microbiome of adolescents - a randomized controlled clinical trial. *PLoS One* 10:e0137318. 10.1371/journal.pone.0137318 26332408 PMC4558009

[B35] Kreth J. Vu H. Zhang Y. Herzberg M. C. (2009). Characterization of hydrogen peroxide-induced DNA release by *Streptococcus sanguinis* and *Streptococcus gordonii*. *J. Bacteriol.* 191 6281–6291. 10.1128/jb.00906-09 19684131 PMC2753043

[B36] Lee E. Park S. Um S. Kim S. Lee J. Jang J. (2021). Microbiome of saliva and plaque in children according to age and dental caries experience. *Diagnostics* 11:1324. 10.3390/diagnostics11081324 34441259 PMC8393408

[B37] Lucchese A. Gherlone E. (2012). Prevalence of white-spot lesions before and during orthodontic treatment with fixed appliances. *Eur. J. Orthod.* 35 664–668. 10.1093/ejo/cjs070 23045306

[B38] Ma S. Ge W. Yan Y. Huang X. Ma L. Li C. (2017). Effects of *Streptococcus sanguinis* Bacteriocin on deformation, adhesion ability, and young’s modulus of *Candida albicans*. *Biomed. Res. Int*. 2017:5291486. 10.1155/2017/5291486 28612025 PMC5458367

[B39] Ma S. Li H. Yan C. Wang D. Li H. Xia X. (2014). Antagonistic effect of protein extracts from *Streptococcus sanguinis* on pathogenic bacteria and fungi of the oral cavity. *Exp. Ther. Med.* 7 1486–1494. 10.3892/etm.2014.1618 24926331 PMC4043591

[B40] Magoc T. Salzberg S. L. (2011). FLASH: Fast length adjustment of short reads to improve genome assemblies. *Bioinformatics* 27 2957–2963. 10.1093/bioinformatics/btr507 21903629 PMC3198573

[B41] Metwalli K. H. Khan S. A. Krom B. P. Jabra-Rizk M. A. (2013). *Streptococcus mutans*, *Candida albicans*, and the human mouth: A sticky situation. *PLoS Pathog.* 9:e1003616. 10.1371/journal.ppat.1003616 24146611 PMC3798555

[B42] Peleg A. Y. Hogan D. A. Mylonakis E. (2010). Medically important bacterial–fungal interactions. *Nat. Rev. Microbiol.* 8 340–349. 10.1038/nrmicro2313 20348933

[B43] Pinheiro L. B. Coleman V. A. Hindson C. M. Herrmann J. Hindson B. J. Bhat S. (2012). Evaluation of a droplet digital polymerase chain reaction format for DNA copy number quantification. *Anal. Chem.* 84 1003–1011. 10.1021/ac202578x 22122760 PMC3260738

[B44] Pusateri C. R. Monaco E. A. Edgerton M. (2009). Sensitivity of *Candida albicans* biofilm cells grown on denture acrylic to antifungal proteins and chlorhexidine. *Arch. Oral. Biol.* 54 588–594. 10.1016/j.archoralbio.2009.01.016 19249746 PMC2693315

[B45] Qudeimat M. A. Alyahya A. Karched M. Behbehani J. Salako N. O. (2021). Dental plaque microbiota profiles of children with caries-free and caries-active dentition. *J. Dent.* 104:103539. 10.1016/j.jdent.2020.103539 33248211

[B46] Richards V. P. Alvarez A. J. Luce A. R. Bedenbaugh M. Mitchell M. L. Burne R. A. (2017). Microbiomes of site-specific dental plaques from children with different caries status. *Infect. Immun.* 85 e106–e117. 10.1128/iai.00106-17 28507066 PMC5520424

[B47] Robertson M. A. Kau C. H. English J. D. Lee R. P. Powers J. Nguyen J. T. (2011). MI paste plus to prevent demineralization in orthodontic patients: A prospective randomized controlled trial. *Am. J. Orthod.Dentofac. Orthop.* 140 660–668. 10.1016/j.ajodo.2010.10.025 22051486

[B48] Rosier B. T. Buetas E. Moya-Gonzalvez E. M. Artacho A. Mira A. (2020). Nitrate as a potential prebiotic for the oral microbiome. *Sci. Rep.* 10:12895. 10.1038/s41598-020-69931-x 32732931 PMC7393384

[B49] Sadeq A. Risk J. M. Pender N. Higham S. M. Valappil S. P. (2015). Evaluation of the co-existence of the red fluorescent plaque bacteria *P. gingivalis* with *S. gordonii* and *S. mutans* in white spot lesion formation during orthodontic treatment. *Photodiagnosis. Photodyn. Ther.* 12 232–237. 10.1016/j.pdpdt.2015.03.001 25813147

[B50] Schoilew K. Ueffing H. Dalpke A. Wolff B. Frese C. Wolff D. (2019). Bacterial biofilm composition in healthy subjects with and without caries experience. *J. Oral. Microbiol.* 11:1633194. 10.1080/20002297.2019.1633194 31275531 PMC6598481

[B51] Simón-Soro A. Guillen-Navarro M. Mira A. (2014). Metatranscriptomics reveals overall active bacterial composition in caries lesions. *J. Oral. Microbiol.* 6:25443. 10.3402/jom.v6.25443 25626770 PMC4247497

[B52] Sun F. Ahmed A. Wang L. Dong M. Niu W. (2018). Comparison of oral microbiota in orthodontic patients and healthy individuals. *Microb. Pathog.* 123 473–477. 10.1016/j.micpath.2018.08.011 30096429

[B53] Tang Z. Xu W. Zhou Z. Qiao Y. Zheng S. Rong W. (2022). Taxonomic and functional alterations in the salivary microbiota of children with and without severe early childhood caries (S-ECC) at the age of 3. *PeerJ.* 10:13529. 10.7717/peerj.13529 35669952 PMC9165595

[B54] Thiyahuddin N. M. Lamping E. Rich A. M. Cannon R. D. (2019). Yeast species in the oral cavities of older people: A comparison between people living in their own homes and those in rest homes. *J. Fungi.* 5:30. 10.3390/jof5020030 31013697 PMC6617379

[B55] Thompson J. Pikis A. (2012). Metabolism of sugars by genetically diverse species of oral Leptotrichia. *Mol. Oral. Microbiol.* 27 34–44. 10.1111/j.2041-1014.2011.00627.x 22230464 PMC3257818

[B56] Travess H. Roberts-Harry D. Sandy J. (2004). Orthodontics. part 6: Risks in orthodontic treatment. *Br. Dent. J.* 196 71–77. 10.1038/sj.bdj.4810891 14739957

[B57] Treerat P. Redanz U. Redanz S. Giacaman R. A. Merritt J. Kreth J. (2020). Synergism between *Corynebacterium* and *Streptococcus sanguinis* reveals new interactions between oral commensals. *ISME. J.* 14 1154–1169. 10.1038/s41396-020-0598-2 32020052 PMC7174362

[B58] Tufekci E. Dixon J. S. Gunsolley J. C. Lindauer S. J. (2011). Prevalence of white spot lesions during orthodontic treatment with fixed appliances. *Angle. Orthod.* 81 206–210. 10.2319/051710-262.1 21208070 PMC8925248

[B59] Valm A. M. (2019). The structure of dental plaque microbial communities in the transition from health to dental caries and periodontal disease. *J. Mol. Biol.* 431 2957–2969. 10.1016/j.jmb.2019.05.016 31103772 PMC6646062

[B60] Wang F. Zhu L. Liu B. Zhu X. Wang N. Deng T. (2018). Noninvasive and accurate detection of hereditary hearing loss mutations with buccal swab based on droplet digital PCR. *Anal. Chem.* 90 8919–8926. 10.1021/acs.analchem.8b01096 29987923

[B61] White T. J. Bruns T. Lee S. Taylor J. (1990). “Amplification and direct sequencing of fungal ribosomal rna genes for phylogenetics,” in *PCR Protocols. A Guide to Methods and Applications*, eds Innis M.A. Gelfand D. H. Sninsky J. J. White T. J. (Cambridge, MA: Academic Press), 315–322.

[B62] Xiao J. Grier A. Faustoferri R. C. Alzoubi S. Gill A. L. Feng C. (2018). Association between oral *Candida* and *Bacteriome* in children with severe ECC. *J. Dent. Res.* 97 1468–1476. 10.1177/0022034518790941 30049240 PMC6262264

[B63] Xiao J. Moon Y. Li L. Rustchenko E. Wakabayashi H. Zhao X. (2016). *Candida* albicans carriage in children with severe early childhood caries (S-ECC) and maternal relatedness. *PLoS One* 11:e0164242. 10.1371/journal.pone.0164242 27741258 PMC5065202

[B64] Xu H. Jenkinson H. F. Dongari-Bagtzoglou A. (2014). Innocent until proven guilty: Mechanisms and roles of *Streptococcus*–Candidainteractions in oral health and disease. *Mol. Oral. Microbiol.* 29 99–116. 10.1111/omi.12049 24877244 PMC4238848

[B65] Xu H. Sobue T. Thompson A. Xie Z. Poon K. Ricker A. (2013). Streptococcal co-infection augments *Candida* pathogenicity by amplifying the mucosal inflammatory response. *Cell Microbiol.* 16 214–231. 10.1111/cmi.12216 24079976 PMC3956708

[B66] Yang F. Dinis M. Haghighi F. He X. Shi W. Chaichanasakul Tran N. (2022). Oral colonization of *Candida albicans* and *Streptococcus mutans* in children with or without fixed orthodontic appliances: A pilot study. *J. Dent. Sci.* 17 451–458. 10.1016/j.jds.2021.07.026 35028070 PMC8739723

[B67] Yun C. Zhiyan L. Chong Z. Jing L. Xin Z. Derui Z. (2019). Illumina-based sequencing analysis of pathogenic microorganisms in dental caries patients of different chinese ethnic groups. *J. Int. Med. Res.* 47 5037–5047. 10.1177/0300060519866939 31516041 PMC6833427

[B68] Zheng Y. Li Z. He X. (2016). Influence of fixed orthodontic appliances on the change in oral *Candida* strains among adolescents. *J. Dent. Sci.* 11 17–22. 10.1016/j.jds.2014.02.001 30894940 PMC6395155

