# Supplementary material for: Candida albicans enriched in orthodontic derived white spot lesions and shaped focal supragingival bacteriome
Source: Front Microbiol. 2023 Jan 24;14:1084850. doi: 10.3389/fmicb.2023.1084850 (PMC9902512; doi:10.3389/fmicb.2023.1084850)
Supplement: Supplementary file 1 [file Data_Sheet_1.docx]

Supplementary Material

# Supplementary Table

**Table S1. Oligonucleotide primer sequences used in this study**

| Target | Primer name | Sequence (5’ -3’ ) |
| --- | --- | --- |
| Fungi | ITS4 | TCCTCCGCTTATTGATATGC |
|  | ITS5: | GGAAGTAAAAGTCGTAACAAGG |
| *Candida albicans* | SAP-F | CTGCTGATATTACTGTTGGTTC |
|  | SAP-R | CCACCAATACCAACGGTATC |

ITS - Internal Transcribed Spacer Region; SAP - Secreted Aspartyl Proteinase.

## 2 Supplementary Figures


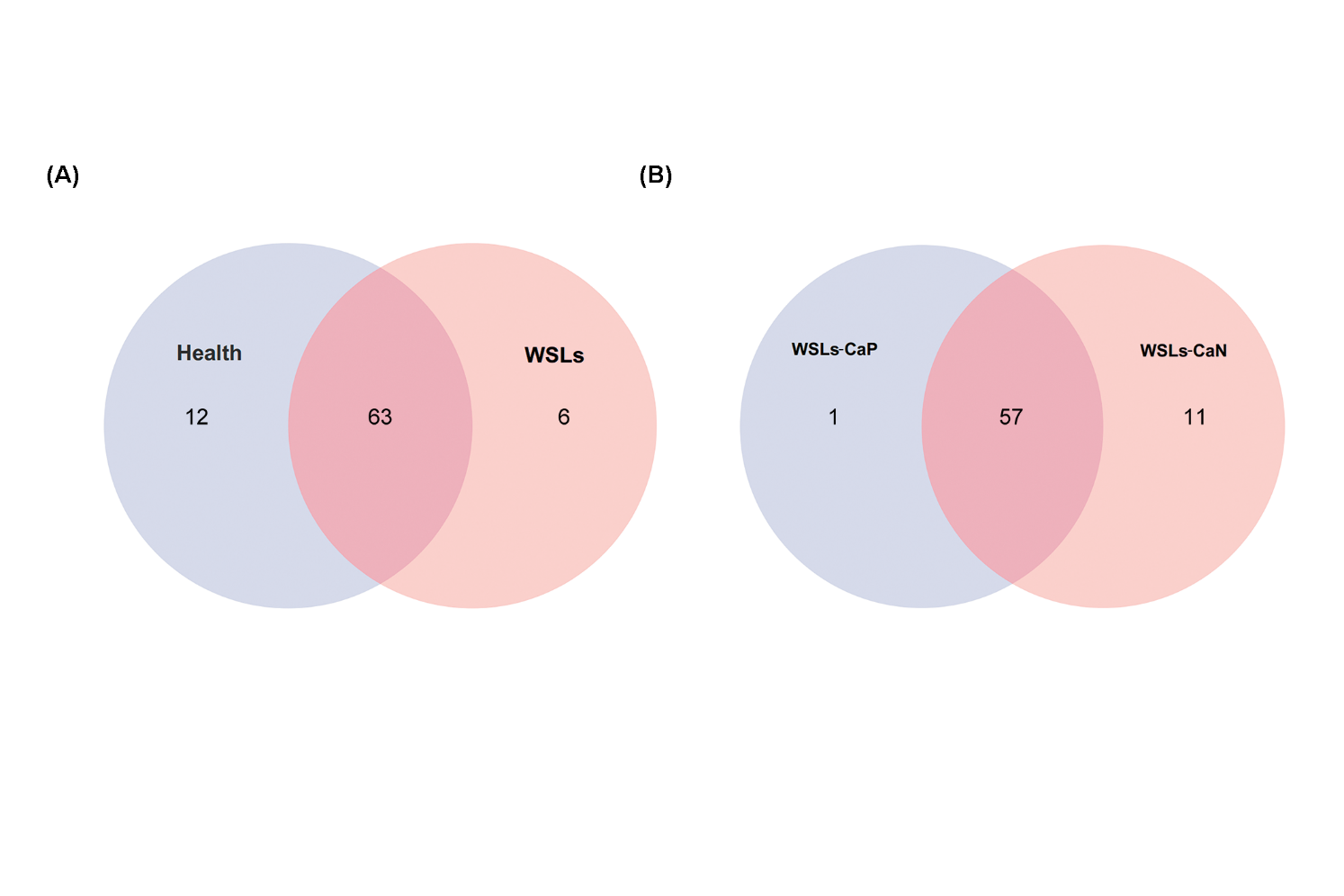


**Supplementary Figure 1.** Comparison of microbial composition via Venn diagram. (**A**) Venn diagram between WSLs and Health group on genus level. Among all annotated genera, 63 genera were uniformly shared by both groups. Twelve genera were uniquely detected in the Health group while 6 genera were in the WSLs group. (**B**) Venn diagram between WSLs-CaP and WSLs-CaN group on genus level. Among all annotated genera, 57 genera were uniformly shared by both groups. Eleven genera were uniquely detected in the WSLs-CaN group while 1 genus was in the WSLs-CaP group.

**
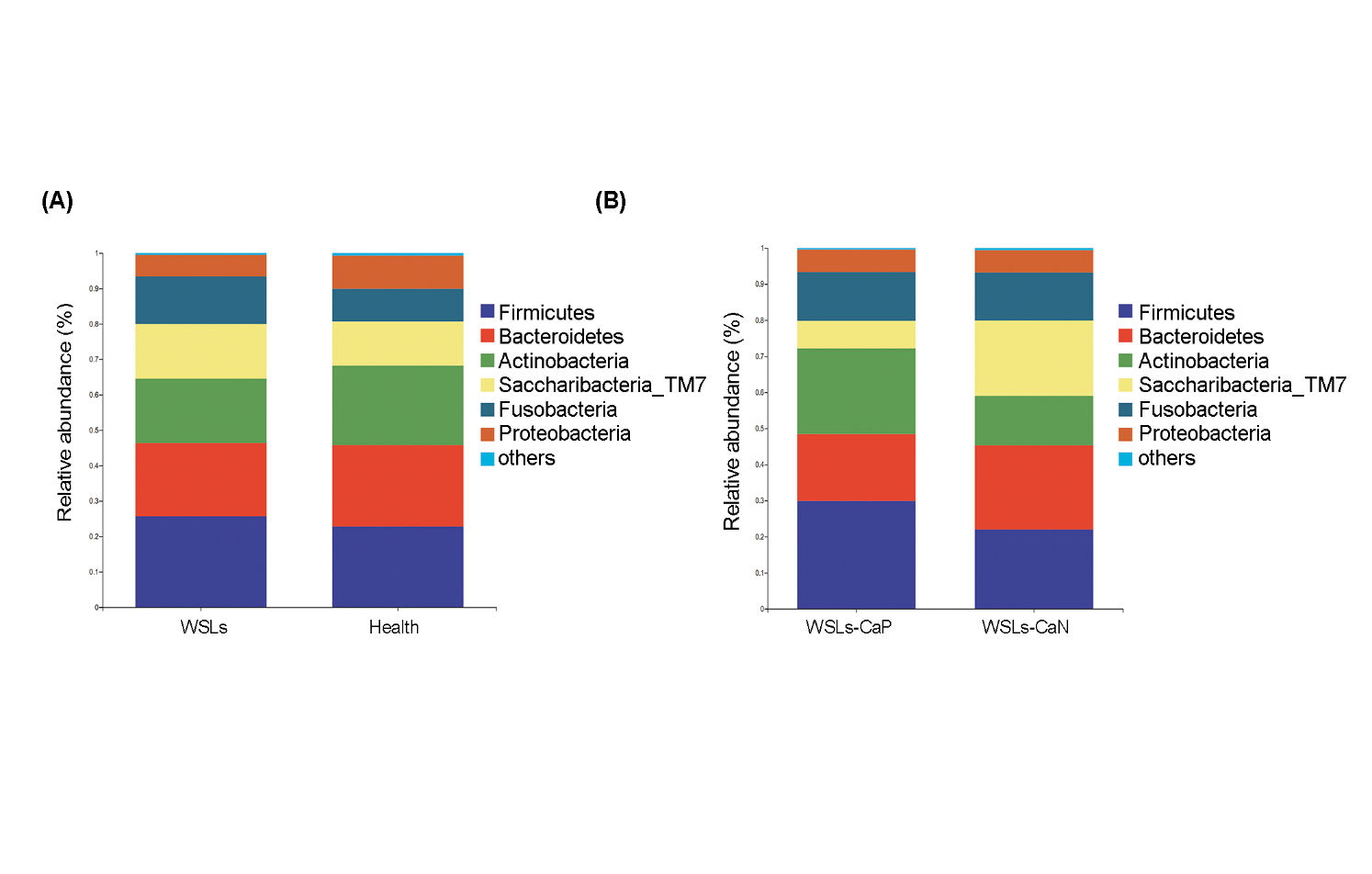
** **Supplementary Figure** **2.** Relative abundances of the core phyla in the detected groups. (**A**) Relative abundances of the core phyla (relative abundance > 1.0%) in WSLs and Health groups. “Others” represented all the phyla less than 1%. (**B**) Relative abundances of the core phyla (relative abundance > 1.0%) in WSLs-CaP and WSLs-CaN groups. “Others” represented all the phyla less than 1%.


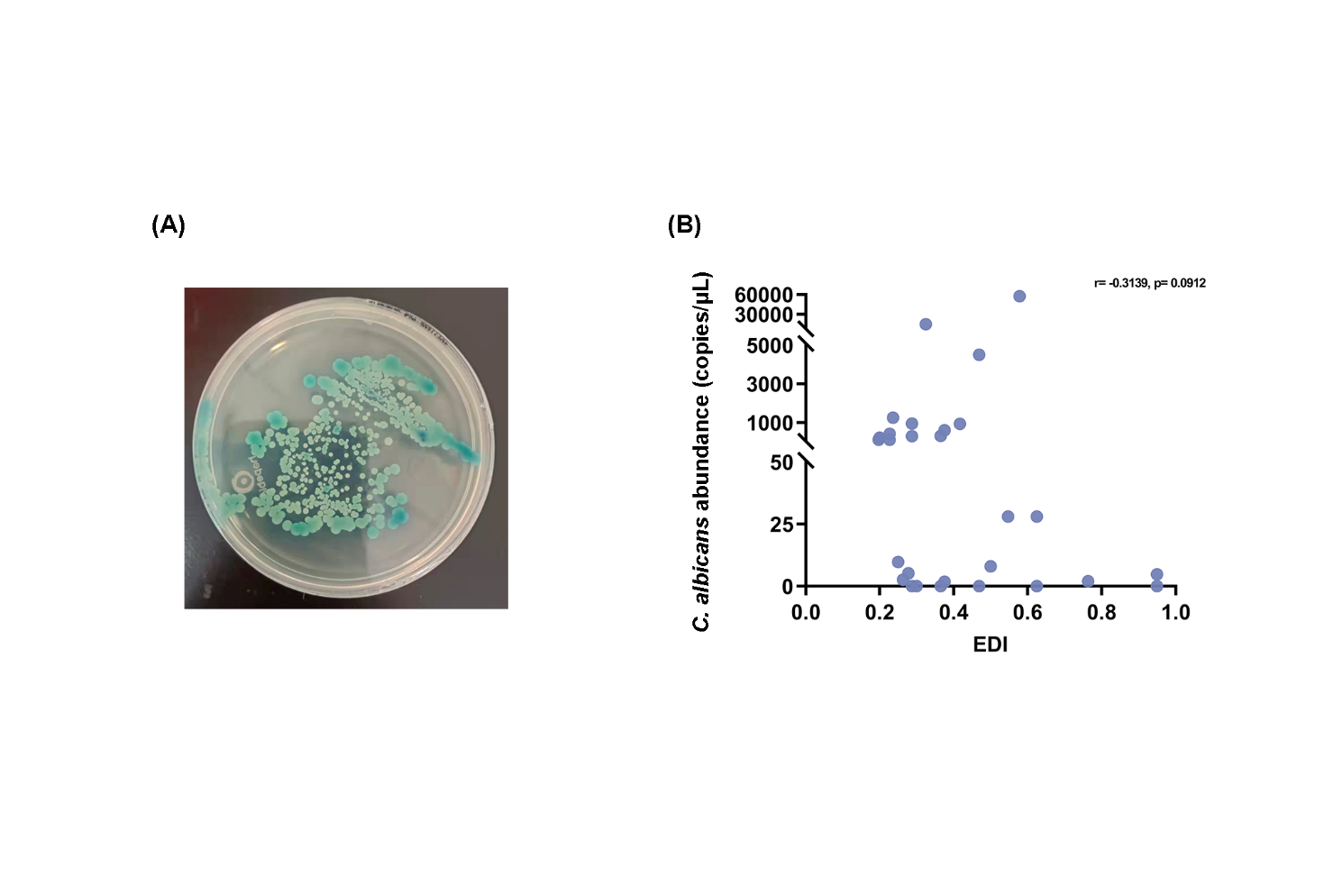


**Supplementary Figure 3.** *C. albicans* cultivation and correlation analysis between *C. albicans* and EDI index. (**A**) Colonies of *C. albicans* cultured on CHROMagar plate at 37 °C for 72 h. *C. albicans* colonies that emerged on CHROMagar showed green colonies. (**B**) The correlation analysis between *C. albicans* and EDI based on Spearman correlation coefficient. In the WSLs group, no significant correlation was detected between EDI and *C. albicans* abundance (P = 0.0919). Although *C. albicans* was significantly enriched in the WSLs group, it did not show a significant correlation with the severity of WSLs.
